# Failure in Double-J stent inserting in laparoscopic pyeloplasty of ureteropelvic junction obstruction: the clinical features and outcomes

**DOI:** 10.1186/s12894-023-01359-7

**Published:** 2023-11-18

**Authors:** Xinyu Wang, Jiayi Li, Songqiao Fan, Zonghan Li, Zhenzhen Yang, Pei Liu, Hongcheng Song, Weiping Zhang

**Affiliations:** Department of Urology, Beijing Children’s Hospital, Capital Medical University, National Center for Children’s Health (NCCH), Beijing, 100045 China

**Keywords:** Children, Double-J stent, Ureteropelvic junction obstruction, Laparoscopic pyeloplasty, Outcome

## Abstract

**Background:**

Double-J (DJ) stent placement is an important procedure during laparoscopic pyeloplasty (LP). Failing to insert the DJ stent may indicate the patient was also complicated with uretero-vesical junction obstruction (UVJO), and surgeons have to change to another alternative drainage method. In the present study, we analyzed the risk factors of failure of DJ stent placement during the LP and reviewed the clinical outcomes of these challenging pyeloplasties.

**Methods:**

We retrospectively analyzed the clinical data of patients with ureteropelvic junction obstruction (UPJO) who underwent LP in our department from January 2016 to September 2020. For patients who developed a difficult process of inserting the DJ stent, the externalized uretero-pyelostomy (EUP) stent was indwelled. Patients were finally divided into two groups: DJ group and EUP group. The primary outcomes were recurrent UPJO, postoperative uretero-vesical junction obstruction (UVJO) and complications.

**Results:**

A total of 535 patients were included in the study, of which 37 patients (6.9%) failed to insert the DJ stent. Age was younger, and weight was lower (*P* < 0.05) in the EUP group. Within follow-up, recurrent UPJO occurred in ten (1.87%) patients, nine in the DJ group and one in the EUP group (*P* > 0.05). The incidence of postoperative UVJO in the EUP group was significantly higher than in the DJ group (10.8% vs. 0.2%, *P* < 0.01). 74 patients (13.8%) developed complications after surgery, 12 patients (32.4%) in the EUP group, significantly higher than that in the DJ group (32.4% vs. 12.4%, *P* < 0.01). Compared with the DJ group, the larger APD were observed in the EUP group at three months postoperatively (3.50 [3.02;4.58] vs. 2.20 [1.50;2.88], *P* < 0.05), but the difference vanished in further follow-up.

**Conclusion:**

The failure of DJ stent placement tends to occur in patients with younger age, lower weight, and larger preoperative APD. Failure may not increase the recurrent UPJO rate, but may indicate a higher probability of postoperative UVJO and may develop more postoperative complications and slower recovery.

## Background

Since Anderson-Hynes dismembered pyeloplasty was advanced in 1949, it has always been the gold standard surgical treatment for ureteropelvic junction obstruction (UPJO) [1]. Recent decades, laparoscopic pyeloplasty (LP) has gradually become a highly-established approach, for it’s minimally-invasive, shorter length of stay(LOS),fewer complications, with similar effects [2].With the development of surgical technique, currently some debates remain on whether it‘s necessary for a urinary stent and the choice of external drainage and internal drainage(Double-J stent) [3]. In recent years, stent-less surgery was recommended to have less complication and similar outcomes [4, 5]. But currently, Double-J(DJ) stent was still the most common choice.

Double-J (DJ) stent placement is an important procedure during LP. Failing to insert the DJ stent happens from time to time for many reasons, most acknowledged of which is indicating the patient was complicated with uretero-vesical junction obstruction (UVJO) [6]. Surgeons have to change to another alternative drainage method. This seem to be a forced resort, whether it influence the outcomes wasn’t clear previously. In the present study, we analyzed the risk factors of failure of DJ stent placement during the LP and reviewed the clinical outcomes of these challenging pyeloplasties. We hypothesized that failure mostly because of stenosis in distal ureter, although the process of treatment was different, it may lead to similar outcomes for pyeloplasty.

## Methods

### Patients

The clinical data of children with primary Andersone-Hynes LP for UPJO were retrospectively reviewed and analyzed between December 2016 to December 2022 in the Department of Urology, Beijing Children’s Hospital. The exclusion criteria are as follows: (1) Patients with a duplex or solitary kidney. (2) Patients combined with vesicoureteral reflux. (3) Patients with bilateral UPJO. (4) Patients with incomplete data or lost to follow-up [7].

UPJO was diagnosed based on the patient’s symptoms and clinical examinations. All patients underwent ultrasound and Intravenous pyelography (IVP) before operation. No evidence of ureteral diameter enlargement were showed in preoperative ultrasound, so that no UVJO was considered. We will consider voiding cystourethrography (VCUG) in children with preoperative ultrasonography indicating ureter dilation or a history of recurrent urinary tract infections (2 or more times). Surgical intervention was recommended when a patient had one or more of the following conditions: (a) Ultrasonography showed progression of hydronephrosis, (b) Patients with symptomatic renal colic, urinary tract infection, and severe upper urinary tract dilatation (Society of Fetal Urology grade III or IV), (c) The renal function of the hydronephrotic kidney is less than 40%. Moreover, the DTPA renogram demonstrated an obstructive pattern (defined as T1/2 > 20 min after administration of furosemide) for reference only. Surgical success was defined as symptom resolution, anteroposterior diameter (APD) decrease, pelvis and caliceal tension decrease in renal ultrasounds, ureters well seen within 40 min in intravenous pyelography or postoperative t1/2 improvement during follow-up [2]. Failure was defined as the recurrent UPJO need to redo dismembered pyeloplasty based on a postoperative obstruction, persistent or worsening hydronephrosis, or symptomatic obstruction.

The patient’s preoperative data, intraoperative parameters, and follow-up information were collected. Preoperative data included patient’s age, sex, weight, preoperative presentation and APD. Intraoperative parameters included operation time, operation side, surgeon and intraoperative drainage. For redo pyeloplasty, restenosis reasons were also collected. Follow-up information mainly included LOS after surgery, recurrent UPJO, postoperative UVJO, and postoperative complications. Postoperative complications were classified according to the Clavien-Dindo classification [8].

### Surgical techniques

Surgical techniques has been described in previous study of our department [7]. All children were positioned supine with the waist elevated and the bed tilted slightly toward the affected side. Cystoscopy was not routinely performed before surgery. LP was performed with three ports (5 mm) transperitoneally. 5 − 0 absorbable monofilament suture was selected. To expose the renal pelvis, colon was mobilized, the adhesions were removed until the UPJ was identified. After UPJ was dissected and the stenotic segment was removed, the DJ stent was first placed in an antegrade fashion. For those with DJ stent well placed firstly, we simultaneously placed perinephric drain and urethral catheter. The appropriate catheter size was selected based on the patient’s age. For patients aged 0–2 years, 2–5 years, 5–10 years, and 10–16 years, 6Fr, 8Fr, 8–10 Fr, and 10–12 Fr catheters were selected, respectively. As for the DJ stent, selection was made according to patients’ age and height, usually 3 F for patients under 2 years old, 4.7 F for patients above, the length of the ureter was calculated based on height [9]. Zebra urological guide-wire wasn’t used intraoperatively.

If the primary attempt was end up with failure and changing to a smaller size still had significant resistance at the ureterovesical junction (UVJ), a situation of difficult process of inserting DJ stent was considered. Then EUP was adopted. The EUP technology is also mentioned in other literature [10]. Preserved the D-J tube as a stent tube in the proximal ureter through the incision of UPJ. A catheter was used as a nephrostomy tube to enter the renal pelvis from the proximal end and drain urine from the renal pelvis. Both the DJ tube and the nephrostomy tube passed through the abdominal wall. All procedures were performed by surgeons with the same qualifications of pyeloplasty surgery [7]. Surgeons were classified into chief physician and associate chief physician groups based on their experience [7].

Postoperatively, oral feeding is given once the patient experiences flatulence, defecation, or reappearance of bowel sounds. The perinephric drain was removed when the remaining output of the drainage increased less than 10 ml within 24 h. The Foley catheter and external ureteral stent were removed before the patient’s discharge. After discharge, prophylactic antibiotic (cephalosporin, 50 mg/kg. d) was maintained for 1–2 weeks. Cystoscopic removal of the DJ stent was done under general anesthesia at 4–6 weeks postoperatively. The nephrostomy tube was removed in accordance with the methylene blue study before discharge, which was usually 10 to 14 days after surgery [7]. In the methylene blue test, only the nephrostomy tube was left and the urinary catheter had been removed. Methylene blue was injected into the nephrostomy tube and the nephrostomy tube was clamped. If there was no distal obstruction, the child would urinate blue urine. After injection, parents were asked to keep on observing the situation of urination. And inform doctor immediately after the blue urine, so as to evaluate the methylene blue experiment situation. Children whose methylene blue tests failed were discharged with a nephrostomy tube. In the follow-up review, we performed nephrostogram under fluoroscopy to determine the shape of the renal pelvis, UPJ, and distal ureter. This was used to assess whether the nephrostomy tube could be removed or a second operation. Routine follow-up for all patients included assessment in the clinic at 3 (after DJ stents removal), 6, 12, 18, and 24 months postoperatively under outpatient review or telephone interview [7].

### Statistical analysis

Statistical analysis was completed by R software (version 4.0.3, http://www.r-project.org). Analyzing by Mann-Whitney U test, median and inter-quartile range was reported for continuous data that did not follow normal distribution, while variables between groups were compared through Chi-square test or Fisher exact test. All statistical results were reported as two-tailed *P* values, < 0.05 is considered statistical significance [7].

## Results

A total of 597 patients underwent LP, 62 patients were excluded according to the exclusion criteria: Ten patients with duplex kidney or solitary kidney, seven patients combined with vesicoureteral reflux, eight patients with bilateral UPJO, and 37 patients with incomplete data or lost to follow-up. Finally, 535 patients underwent were further included in the study. Patients were divided into two groups according to whether they failed to insert the DJ stent during the operation (DJ group and EUP group).

### Patients’ characteristics between DJ group and EUP group

A total of 535 patients were included in the study, all patients were Society for Fetal Urology (SFU) grade IV. 498 patients insert the DJ stent (DJ group), and 37 patients (6.9%) failed to insert the DJ stent during the operation (EUP group). The median age of the DJ group was 53.3[22.7;95.2] months, significantly older than that in the EUP group (24.6 [17.4;48.9] month, *P* < 0.001), and the median weight of the DJ group was significantly higher than that in the EUP group (18.0 [12.4;26.0] vs. 13.0 [11.0;17.3]kg, *P* < 0.001). 225 patients (45.2%) in the DJ group presented initially with flank pain, nausea or vomiting, and associated hydronephrosis, significantly more than that in the EUP group (5 patients, 13.5%, *P* < 0.01). No ureteral dilatation was observed on preoperative ultrasound in the DJ group and the EUP group. The preoperative APD in the DJ group was smaller than that in the EUP group (2.80 [2.20;3.60] vs. 3.40 [2.50;4.40] cm, *P* = 0.003). The median operative time of the DJ group was 110 [83.0;139] min, which is significantly shorter than that in the EUP group (113[100;158] min, *P* = 0.020). Postoperative LOS after surgery in the DJ group was 6.00 [6.00;7.00] days, shorter than that in the EUP group (9.00 [8.00;11.00] days *P* < 0.001).

### Clinical outcomes between DJ group and EUP group

The follow-up time ranged from 14.2 to 78 months. As shown in Table 1 and 12 patients (32.4%) in the EUP group developed complications, significantly higher than that in the DJ group (62 patients, 12.4%, *P* = 0.002). The details were showed in Table 2. In the DJ group, 32 patients (6.43%) developed febrile UTI after DJ stent removal and relieved after receiving antibiotics therapy (Clavien II). Four patients (0.80%) developed intestinal paralyzes and relieved after fasting plus gastrointestinal decompression (Clavien II). Three patients developed UPJ leakage requiring percutaneous nephrostomy (Clavien IIIb). Besides, four patients (0.80%) underwent debridement and suturing because of hernia formation (Clavien IIIb), and four (0.80%) underwent debridement and reclosure because of delayed wound healing (Clavien IIIb). Perirenal abscess formation was observed in one patient (0.20%), he underwent the nephrostomy (Clavien IIIb). DJ stent migration occurred in four patients (0.80%), we reset the DJ stent (Clavien IIIb). In the EUP group, three patients (8.11%) developed febrile UTI after nephrostomy tube removal and relieved after receiving antibiotics therapy (Clavien II). Prolapse (included displacement or extrusion) of the nephrostomy tube occurred in two patients (5.41%), and they underwent the nephrostomy (Clavien IIIb). Two patients (5.41%) exhibited poor wound healing around the fistula and received fistula extraction, debridement and reclosure (Clavien IIIb).

**Table 1:** Patients characteristics between DJ group and EUP group

	DJ group (*n* = 498)	EUP group (*n* = 37)	*P* value
**Sex (Male/Female)**	409/89	33/4	0.385
**Age (m)**	53.3 [22.7;95.2]	24.6 [17.4;48.9]	**0.004**
**Weight (kg)**	18.0 [12.4;26.0]	13.0 [11.0;17.3]	**< 0.001**
**Kidney malformation (Y/N)**	10/488	3/34	0.054
**Preoperative presentation (Y/N)**	225/273	5/32	**< 0.001**
**Abdominal surgery history (Y/N)**	11/487	0/37	1.000
**Operation side (L/R)**	387/111	26/11	0.402
**Surgeon (Chief/Associate chief)**	168/329	12/25	1.000
**Preoperative APD (cm)**	2.80 [2.20;3.60]	3.40 [2.50;4.40]	**0.003**
**Obstruction reason**			0.641
** UPJ stenosis**	432 (86.7%)	31 (83.8%)	
** Ectopic vascular**	29 (5.82%)	2 (5.41%)	
**Others**	37 (7.43%)	4 (10.8%)	
**Operation time (min)**	110 [83.0;139]	113 [100;158]	**0.020**
**Length of stay (d)**	6.00 [6.00;7.00]	9.00 [8.00;11.0]	**< 0.001**
**Complications (Y/N)**	62/436	12/25	**0.002**
**Recurrent UPJO (Y/N)**	9/489	1/36	0.515
**Postoperative UVJO (Y/N)**	1/497	4/33	**< 0.001**
**Post-operatively APD(cm)**			
**3 months**	2.20 [1.50;2.88]	3.50 [3.02;4.58]	**0.027**
**6 months**	1.60 [1.15;2.20]	1.95 [1.42;2.45]	0.179
**12 months**	1.60 [1.17;2.10]	1.85 [1.33;2.70]	0.092
**18 months**	1.70 [1.35;2.25]	1.90 [1.50;3.30]	0.509
**24 months**	1.60 [1.15;2.15]	1.75 [1.37;2.80]	0.195

Bold indicates statistical significance

*DJ *Double-J, *EUP *externalized uretero-pyelostomy, *UPJ *ureteropelvic junction, *UPJO *ureteropelvic junction obstruction, *UVJO *uretero-vesical junction obstruction

**Table 2:** Postoperative complications between DJ group and EUP group

Complications	Clavien Dindo grading	DJ group, n (%)	EUP group, n (%)
**febrile UTI**	II	32 (6.43%)	3 (8.11%)
**Intestinal paralysis**	II	4 (0.80%)	/
**UPJ leakage**	IIIb	3 (0.60%)	
**Hernia formation**	IIIb	4 (0.80%)	/
**Prolapse of nephrostomy tube**	IIIb	/	2 (5.41%)
**Delayed wound healing**	IIIb	4 (0.80%)	2 (5.41%)
**Perirenal abscess formation**	IIIb	1 (0.20%)	/
**DJ stent migration**	IIIb	4 (0.80%)	/
**Postoperative UVJO**	IIIb	1 (0.20%)	4 (10.8%)
**Recurrunt UPJO**	IIIb	9 (1.81%)	1 (2.70%)

*DJ *Double-J, *EUP *externalized uretero-pyelostomy, *UTI *urinary tract infection, *UPJ *ureteropelvic junction, *UPJO *ureteropelvic junction obstruction, *UVJO *uretero-vesical junction obstruction

Recurrent UPJO was observed in nine patients (1.81%) in the DJ group and one patient (2.70%) in the EUP group during the follow-up. They underwent redo pyeloplasty. The Recurrent UPJO rates were Not statistically significant between these two groups (*P* > 0.05). Four patients (10.8%) developed postoperative UVJO in the EUP group, significantly higher than that in the DJ group (one patients, 0.20%, *P* < 0.001), they underwent ureteral reimplantation. These children had clamped fistulae or had fever after fistulae ejection, without pain or vomiting.

### Characteristics of APD between DJ group and EUP group

**Fig. 1:**
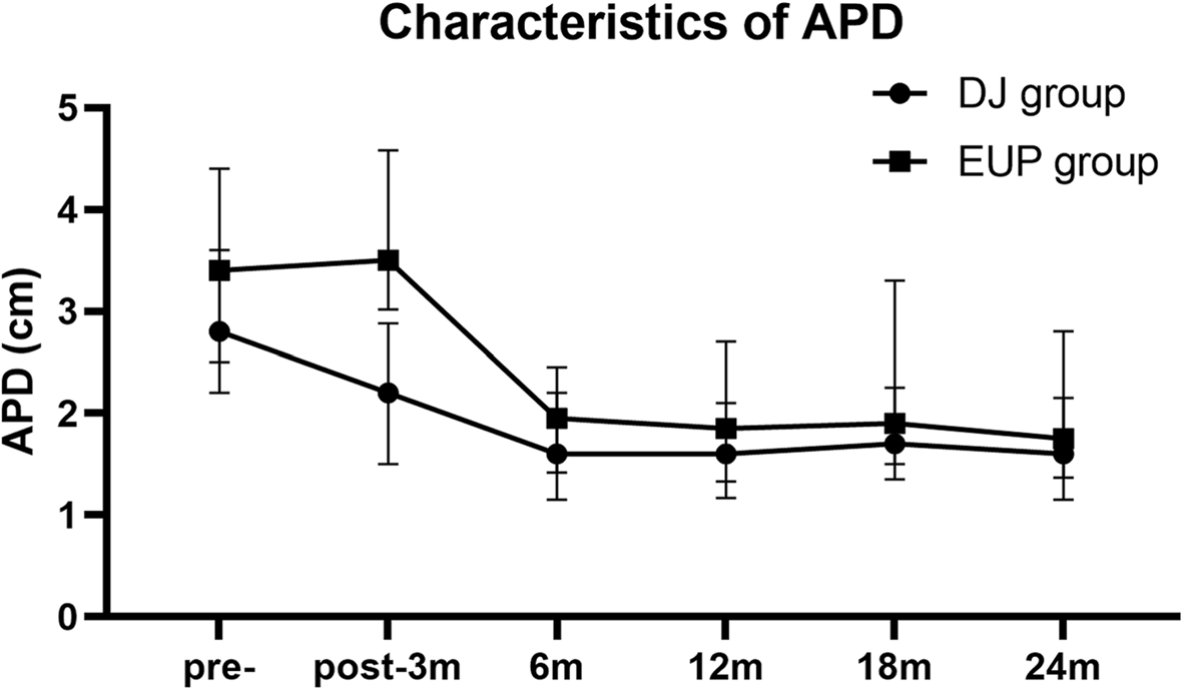
Changes in anteroposterior diameter (APD) during pre- and post-operative periods.  The preoperative APD in the DJ group was smaller than that in the EUP group (2.80 [2.20;3.60] vs. 3.40 [2.50;4.40] cm, *P *= 0.003). During follow-up, we found significant differences at three months postoperatively, of which APD in the EUP group is larger than that in the DJ group (2.20 [1.50;2.88] vs. 3.50 [3.02;4.58]cm, *P *= 0.027). The difference vanished at six months, 12 months and 24 months after surgery (*P* > 0.05).

The follow-up time ranged from 14.2 to 78 months, patients demonstrated an improvement in hydronephrosis. Figure 1 depicts the changes in APD during preoperative and postoperative periods between the DJ group and the EUP group. The preoperative APD in the DJ group was smaller than that in the EUP group (2.80 [2.20;3.60] vs. 3.40 [2.50;4.40] cm, *P* = 0.003). At three months postoperatively, APD in the EUP group is significantly larger than that in the DJ group (2.20 [1.50;2.88] vs. 3.50 [3.02;4.58] cm, *P* = 0.027). At six months postoperatively, the APD in the EUP group is 1.85 [1.33;2.70] cm, higher than that in the DJ group (1.60 [1.17;2.10] cm), but the difference is not significant (*P* = 0.179). At one year postoperatively, the APD in the EUP group is 1.90 [1.50;3.30] cm, the APD in the DJ group is 1.70 [1.35;2.25] cm, the difference is not significant (*P* = 0.092). At 18 months postoperatively, the APD in the EUP group is 1.90 [1.50;3.30] cm, the APD in the DJ group is 1.70 [1.35;2.25] cm, the difference is not significant (*P* = 0.509). At 2 years postoperatively, the APD in the EUP group is 1.75 [1.37;2.80] cm, the APD in the DJ group is 1.60 [1.15;2.15]cm, the difference is not significant (*P* = 0.195).

## Discussion

LP for UPJO was developed in 1990s, as a well-accepted surgical procedure, the use of stent drainage remains controversial [11, 12]. Comparing to externalized pyelo-ureteral stents or stent-less, although a second general anesthesia was required and risk of migration exist, DJ stent was most widely used in LP, for it’s easier to insert, with the highest operative success, renal function improvement, and the shortest hospital stay [13, 14]. But lack of study research the reason and outcome of failure in DJ stent inserting. In present study, we tried to sum the clinical features and outcomes of the unexpected procedure.

The process of surgery and definition of failure was standardized clearly. Changing size of DJ stent timely minimized the probability of iatrogenic injury. There is currently no uniform standard practice of preoperative retrograde pyelography (RPG). Cockrell and Hendren reported that of the 100 children who underwent pyeloplasty, 36 had RPGs showing stenosis other than UPJO [15]. Nicole Golda et al. encouraged routine preoperative RPG practice, as they thought that can help clear the type and location of abnormalities, which can assist in the development of surgical plans [16]. While Rushton et al. [17] and Bachor et al. [18] argued that RPG should not routinely performed before pyeloplasty. In our department, none RPG was done preoperatively, as we considered that most UVJO can be diagnosed through preoperative ultrasound, and RPG may show abnormal result in a number of normal patients without UVJO, besides the risk of urinary tract infection.

The baseline data of two groups was shown in Table 1, trying to figure out the clinical features of patients tend to fail in DJ stent inserting. It showed that the age and weight of EUP group was significantly lower than DJ group. There were studies proposed that LP has high success with low rate of complications in low-weight(≤ 10 kg) and young-age(less than 2 years old)children [19, 20]. But previous study in our department revealed an contrary conclusion, that low weight and age played a negative role in LP [21]. Present result proved our previous opinion, that fail of DJ stent inserting usually happened in children who were younger with lower weight. We supposed that may due to the limited space for surgical maneuvers, smaller size of ureter and UVJ and less tolerance to manipulations. Otherwise, the preoperative APD was higher in the EUP group, indicating a greater severity.

We noticed that the postoperative recurrence rate of UPJO in the DJ group was 1.81%, and in the EUP group was 2.70%, with no significant difference in success rate (*P* > 0.05). Several studies comparing the two ways of drainage were achieved. Sarhan [22] revealed that the success rate of UPJO in children using DJ tube as internal drainage was 95.5%, for children with external drainage was 97%, no significant differences were detected. In a meta-analysis of 839 children from 8 studies by Liu et al. [14], the mean success rates in the DJ stent group and the EUP group were 93.2% (range: 88-95%) and 92.6% (range: 86-94.7%), with no significant difference. Our study showed that even if the DJ tube failed to be inserted intraoperatively, switching to the external drainage method didn’t affect the success rate of the pyeloplasty either.

For children who failed in DJ stent insert, suspicion of the concurrent presence of UVJO was necessary. In a study that reviewed 254 children with UPJO over 8 years, Halder et al. [6] found 5 children with both UPJO and UVJO. These 5 children were failed to insert the DJ stent intraoperatively. Postoperative nephrostogram confirmed the diagnosis. Lee et al. [23] reviewed 447 children in a 10-year study and found a total of 15 children with both UPJO and UVJO, 10 of whom were diagnosed preoperatively, and all of them had particularly severe Obstructive manifestations. In 4 cases, only UPJO was diagnosed because no obvious dilated ureter was found in preoperative examination, and UVJO was found during operation. Only UVJO was diagnosed preoperatively in another 1 case. Children with UPJO and UVJO at the same time are relatively rare, and it is sometimes difficult to detect both at the same time through preoperative examination. In this case, intraoperative diagnosis is particularly important. In present study, 1/497 of the children with undiagnosed UVJO intraoperatively were found in the DJ stent group, while 4/33 of the children in the EUP group were found after surgery, which was much higher than the DJ stent group, and the difference was statistically significant (*P* < 0.05). Despite the DJ tube can pass through, there are still nearly 10% of children with UVJO. This shows that the intraoperative DJ stent placement is difficult, which may reflect the existence of UVJO to a certain extent, but the overall proportion is not high. These children recovered after the second operation. The remaining children were not found to have combined UVJO in the postoperative follow-up, indicating that other factors may still lead to the occurrence of this situation. We speculate that the reason of failure in the remaining 29/33 of children without UVJO may be related to the intraoperative manipulations. The placement of the DJ stent, especially when the size is inappropriate, may cause minor damage to the distal ureter, resulting in transient distal ureteral edema, which hinders our operation. Repeated attempts of DJ stent placement may aggravate the process. We observed that from the end of the operation to the second day after the operation, the DJ tube changed to the external drainage stent would have a large amount of urine leakage, but no more urine leakage after long-term postoperatively. At the same time, after the removal of the DJ tube, there was no recurrence of hydronephrosis, which may indicate that the transient edema of the distal ureter subsided.

It might be difficult to diagnose coexisting UPJO and UVJO, or other distal obstruction correctly. The retrograde placement of a DJ tube by cystoscopy can indeed be used to determine whether the patient has concurrent distal obstruction. That fit open surgery more, while for laparoscopy, it was more easier to find out the position of obstruction. Besides, for those patients who had UPJO accompany with UVJO, procedures on the UPJ and UVJ are not generally recommended because of the distribution of ureteral vessels. In Campbell Urology Surgery, UVJO has the possibility of self-healing. Therefore, it is feasible to perform UPJO operation first and then follow up to observe the condition of UVJO in children. In our center, we prefer to treat UPJO first. And cystoscopy and retrograde stenting require the adjustment of position, which was not chosen. EUP is used for children who fail to place DJ tubes. It may help alleviate symptoms and provide a buffer time for possible self-healing if the nephrostomy tube cannot be removed at discharge.

For the postoperative complication rate, the EUP group was significantly higher than that in the DJ group (*P* = 0.02). Chu et al. [4] suggested that the negative effect of EUP approach mainly manifested as urine leakage, damage to the renal parenchyma, skin site infection and delayed wound healing. In the current study, 3 patients experienced urine leakage, 1 was in the DJ group and 2 were in the EUP group. While 2 patients had postoperative skin infection around the stoma and led to delayed wound healing. However, DJ stent was related to a higher risk of UTI and requires additional general anesthesia to remove the stent [24]. In addition, Methylene blue testing is routinely performed during postoperative hospitalization in patients with EUP drainage, in order to assess whether the anastomosis is free, the success of surgery can be judge clearly in hospitalization.

As comparing the postoperative ultrasound parameters of two groups, only the APD of three-months postoperative was significant different. The recovery of APD was faster in the DJ stent group than that in the EPU group. Consistent with the study designed Fernandez-Ibieta et al. [25], children could still have a larger APD at 3 months postoperatively. There are usually large changes within 6 months and tend to plateau after that. It may be due to the presence of a certain degree of edema at the anastomotic stoma postoperative within 3 months. In the EPU group, without support for the anastomosis, may lead to a slower growth rate and slower edema subsidence than the DJ tube group. However, there was no significant difference in APD between the DJ stent group and the EPU group at 6 months and longer follow-up.

Limitations should be considered in this study. First, since it was a retrospective study from single center, may lead to potential selection bias. Secondly, lack of evaluation of renal function, such as radionuclide renal dynamic imaging, and the long period span and irregular outpatient review may lead to loss of clinical data. Third, as mentioned before, we didn’t perform RPG routinely, miss diagnosed of UVJO was possible. Besides, the average LOS of EUP group in our department was longer than previous study, as we intended to ensure the success through Methylene blue testing before discharge. Finally, the low number of complications may limit comparisons and not show differences between DJ and EUP groups.

## Conclusion

For patients who failed to insert DJ stents during the LP, selecting a suitable drainage method is challenging. Our study showed that the failure of DJ stent placement tends to occur in patients with younger age, lower weight, and larger preoperative APD. Although the failure of DJ stent placement may not increase the recurrent UPJO rate, it may indicate a higher probability of postoperative UVJO and may develop more postoperative complications and slower recovery. Patients who failed to insert the DJ stent during the operation should be monitored.

## Data Availability

The datasets used and/or analyzed during the current study are available from the corresponding authors and first authors on reasonable request.

## Abbreviations

DJ stent: Double-J stent
UPJO: Ureteropelvic junction obstruction
LP: Laparoscopic pyeloplasty
LOS: Length of stay
IVP: Intravenous pyelography
VCUG: Voiding cystourethrography
VUR: Vesicoureteral reflux
UVJO: Uretero-vesical junction obstruction
APD: Anteroposterior diameter
EUP: Externalized uretero-pyelostomy

## References

[1] O’Reilly PH, Brooman PJ, Mak S (2001). The long-term results of Anderson-Hynes pyeloplasty. BJU Int.

[2] Mei H, Pu J, Yang C, Zhang H, Zheng L, Tong Q (2011). Laparoscopic versus open pyeloplasty for ureteropelvic junction obstruction in children: a systematic review and meta-analysis. J Endourol.

[3] Yeung CK, Tam YH, Sihoe JD, Lee KH, Liu KW (2001). Retroperitoneoscopic dismembered pyeloplasty for pelvi-ureteric junction obstruction in infants and children. BJU Int.

[4] Kim J, Park S, Hwang H (2012). Comparison of Surgical outcomes between Dismembered Pyeloplasty with or without Ureteral Stenting in Children with Ureteropelvic Junction obstruction. Korean J Urol.

[5] Bilen CY, Bayazit Y, Güdeloğlu A, Abat D, Inci K, Doran S (2011). Laparoscopic pyeloplasty in adults: stented versus stentless. J Endourol.

[6] Halder P, Shukla RM, Mandal KC, Mukhopadhyay B, Barman S (2014). Double obstruction of ureter: a diagnostic challenge. J Indian Assoc Pediatr Surg.

[7] Li J, Yang Y, Li Z (2022). Redo laparoscopic pyeloplasty for recurrent ureteropelvic junction obstruction: propensity score matched analyses of a high-volume center. Front Pediatr.

[8] Clavien PA, Barkun J, de Oliveira ML (2009). The Clavien-Dindo classification of Surgical Complications: five-year experience. Ann Surg.

[9] Palmer JS, Palmer LS (2007). Determining the proper stent length to use in children: age plus 10. J Urol.

[10] Helmy T, Blanc T, Paye-Jaouen A, El-Ghoneimi A (2011). Preliminary experience with external ureteropelvic stent: alternative to double-j stent in laparoscopic pyeloplasty in children. J Urol.

[11] Penn HA, Gatti JM, Hoestje SM, DeMarco RT, Snyder CL, Murphy JP (2010). Laparoscopic versus open pyeloplasty in children: preliminary report of a prospective randomized trial. J Urol.

[12] Braga LHP, Lorenzo AJ, Farhat WA, Bägli DJ, Khoury AE, Pippi Salle JL (2008). Outcome analysis and cost comparison between externalized pyeloureteral and standard stents in 470 consecutive open pyeloplasties. J Urol.

[13] Lee LC, Kanaroglou N, Gleason JM (2015). Impact of drainage technique on pediatric pyeloplasty: comparative analysis of externalized uretero-pyelostomy versus double-J internal stents. Can Urol Assoc J.

[14] Liu X, Huang C, Guo Y, Yue Y, Hong J (2019). Comparison of DJ stented, external stented and stent-less procedures for pediatric pyeloplasty: a network meta-analysis. Int J Surg.

[15] Cockrell SN, Hendren WH (1990). The importance of visualizing the ureter before performing a pyeloplasty. J Urol.

[16] Golda N, Kapoor A, DeMaria J (2008). Laparoscopic pyeloplasty: role of preoperative retrograde pyelography. J Pediatr Urol.

[17] Rushton HG, Salem Y, Belman AB, Majd M (1994). Pediatric pyeloplasty: is routine retrograde pyelography necessary?. J Urol.

[18] Bachor R, Kleinschmidt K, Gottfried HW, Hautmann R (1997). [Is retrograde ureteropyelography necessary before kidney pelvis-plasty in childhood?]. Urologe A.

[19] Neheman A, Noh PH, Piaggio L, González R (2008). The role of laparoscopic Surgery for urinary tract reconstruction in infants weighing less than 10 kg: a comparison with open Surgery. J Pediatr Urol.

[20] Badawy H, Saad A, Fahmy A (2017). Prospective evaluation of retroperitoneal laparoscopic pyeloplasty in children in the first 2 years of life: is age a risk factor for conversion?. J Pediatr Urol.

[21] He Y, Song H, Liu P (2020). Primary laparoscopic pyeloplasty in children: a single-center experience of 279 patients and analysis of possible factors affecting Complications. J Pediatr Urol.

[22] Sarhan O, Al Awwad A, Al Otay A (2021). Comparison between internal double J and external pyeloureteral stents in open pediatric pyeloplasty: a multicenter study. J Pediatr Urol.

[23] Lee YS, Im YJ, Lee H (2014). Coexisting ureteropelvic junction obstruction and ureterovesical junction obstruction: is pyeloplasty always the preferred initial Surgery?. Urology.

[24] Elmalik K, Chowdhury MM, Capps SNJ (2008). Ureteric stents in pyeloplasty: a help or a hindrance?. J Pediatr Urol.

[25] Fernández-Ibieta M, Nortes-Cano L, Guirao-Piñera MJ, Zambudio-Carmona G, Ruiz-Jiménez JI (2016). Radiation-free monitoring in the long-term follow-up of pyeloplasty: are ultrasound new parameters good enough to evaluate a successful procedure?. J Pediatr Urol.

